# Association of chorioretinal thickness with chronic kidney disease

**DOI:** 10.1186/s12886-023-02802-x

**Published:** 2023-02-09

**Authors:** Ahmed Ibrahim Basiony, Sherry Nissem Atta, Noha Mohamed Dewidar, Adel Galal Zaky

**Affiliations:** 1 Ophthalmology, Menoufia Faculty of Medicine, Yassin Abdelghaffar St., ShebinElkom, Menoufia 32511 Egypt; 2 Ophthalmology, Menoufia Faculty of Medicine, ShebinElkom, Egypt; 3 Nephrology, Menoufia Faculty of Medicine, ShebinElkom, Egypt

**Keywords:** Chronic kidney disease, Optical coherence tomography, Retina, Retinal nerve fiber layer, Choroid

## Abstract

**Objective:**

To assess retinal and choroidal thickness changes in chronic kidney disease (CKD) patients using spectral domain optical coherence tomography (SD-OCT).

**Background:**

CKD is a devastating health trouble. The eye and the kidney share similar structural and genetic pathways, so that kidney disease and ocular disease may be closely linked. OCT is a precise, fast method for high-definition scanning of the retina and choroid.

**Patients and methods:**

A cross sectional study was conducted at Menoufia University Hospital ophthalmology department on 144 eyes of 72 CKD patients divided into 3 groups according to the stage of CKD as follows: group 1: CKD stage 1–2, with Glomerular Filtration Rate (GFR) > 60 ml/min/1.73m^2^ group 2: CKD stage 3, GFR 30–59 ml/min/1.73m^2^ and group 3: CKD stage 4–5, eGFR < 29 ml/min/1.73m^2^. All patients underwent full ophthalmologic examination followed by OCT assessment of retinal, retinal nerve fiber layer (RNFL) and choroidal thickness.

**Results:**

Retinal and choroidal thickness were reduced in group 2 (CKD stage 3) and group 3 (CKD stage 4–5) compared with group 1 (CKD stage 1–2). The reduction was more severe in group 3 than group 2. RNFL thickness did not differ between groups. A thinner retina and choroid were associated with an elevated serum C-reactive protein (CRP) concentration, and greater degrees of proteinuria.

**Conclusion:**

Chorioretinal thinning in CKD is associated with a lower eGFR, a higher CRP, and greater proteinuria. Further studies, in a large scale of patients, are needed to detect whether these eye changes reflect the natural history of CKD.

## Introduction

Chronic kidney disease (CKD) is a significant health concern that affects a large portion of the population, leading to a considerable economic burden and reduced quality of life [1]. The severity of the disease is classified into stages based on the level of reduction in GFR. Stage 1 is characterized by a slight reduction in kidney function with normal or relatively high GFR (> 90 ml/min/1.73 m^2^) and persistent albuminuria. Stage 2 is characterized by a mild reduction in GFR (60–89 ml/min/1.73 m^2^), while stage 3 is characterized by a moderate reduction in GFR (30–59 ml/min/1.73 m^2^). Stage 4 is characterized by a severe reduction in GFR (15–29 ml/min/1.73 m^2^), and preparation for kidney replacement therapy is necessary. Stage 5, also known as end-stage kidney disease, is characterized by established kidney failure, requiring permanent kidney replacement therapy [2].

CKD not only affects the kidneys but also other organs in the body. The kidney and eye are strikingly similar in their developmental and pathogenic pathways. The renal podocyte, a stromal cell that supports vasculature and serves as a progenitor of interstitial myofibroblasts in renal fibrogenesis, is structurally and functionally similar to the vascular pericyte in the eye. As a result, diseases that affect one may also affect the other. Podocytes interact with endothelial cells through distinct signaling pathways, and their activation and detachment from capillaries after acute or chronic kidney injury may be critical for driving CKD progression [3].

Retinal and choroidal changes have been observed in patients with CKD. Fundus examination is a valuable tool for visualizing the chorioretinal microvasculature that may be affected in CKD [4]. Optical coherence tomography (OCT), a non-invasive method for imaging the retina and choroid, is a valuable tool for identifying these changes. Spectral domain OCT (SD-OCT) in combination with an enhanced depth imaging (EDI) feature enables high-resolution delineation of different retinal layers, as well as the deeper choroid. These imaging techniques can identify architectural changes within the retina and choroid in CKD patients [5].

The aim of this study is to report retinal and choroidal thickness in association with renal function in patients with CKD. Furthermore, the study will be valuable for identifying patients at risk for developing CKD and for monitoring the progression of the disease. In so doing, we aim to provide a deeper understanding of the relationship between CKD and chorioretinal changes, paving the way for new strategies for early diagnosis and treatment of this prevalent disease.

## Patients and methods

This cross-sectional study involved 144 eyes of 72 CKD patients who are over 18 years of age. Exclusion criteria include eyes with previous laser or surgical treatment, high myopia, corneal diseases, lens, and vitreous opacities that prevent fundus visualization, uveitis, glaucoma or other retinal diseases, and diabetes mellitus. The study was conducted at the department of Ophthalmology, Menoufia University Hospital from November 2020 to March 2021. All study procedures were carried out and approved by the Ethical Committee of Menoufia Faculty of Medicine and in accordance with the declaration of Helsinki. Written informed consent was obtained from each patient before his or her enrollment in the study.

The study included a full systemic and ocular examinations for each subject. Ocular examination included best-corrected visual acuity (BCVA, LogMAR), anterior segment examination using slit lamp biomicroscopy, intraocular pressure (IOP) by Goldman applanation tonometry, and fundus examination using 90-diopters lens. Renal function is assessed through serum creatinine values, estimated glomerular filtration rate (eGFR) calculated using the Chronic Kidney Disease Epidemiology Collaboration (CKD-EPI) equation, mean arterial blood pressure, body mass index (BMI), complete blood count, random blood sugar, traditional C-reactive protein (CRP), erythrocyte sedimentation rate (ESR), serum albumin, and urine analysis.

Retinal and choroidal thicknesses were obtained using SD-OCT (Heidelberg engineering, Heidelberg, Germany) with EDI mode activated. OCT was scheduled to be performed in the morning (around 10–11 a.m) to minimize any potential diurnal variations in the measured choroidal thicknesses across the subjects. Tropicamide 1% eye drops achieved pupillary dilation in all patients. For patients on hemodialysis, OCT was obtained 24 hours after hemodialysis to allow recovery from post-dialysis fatigue and blood volume changes.

**Fig. 1:**
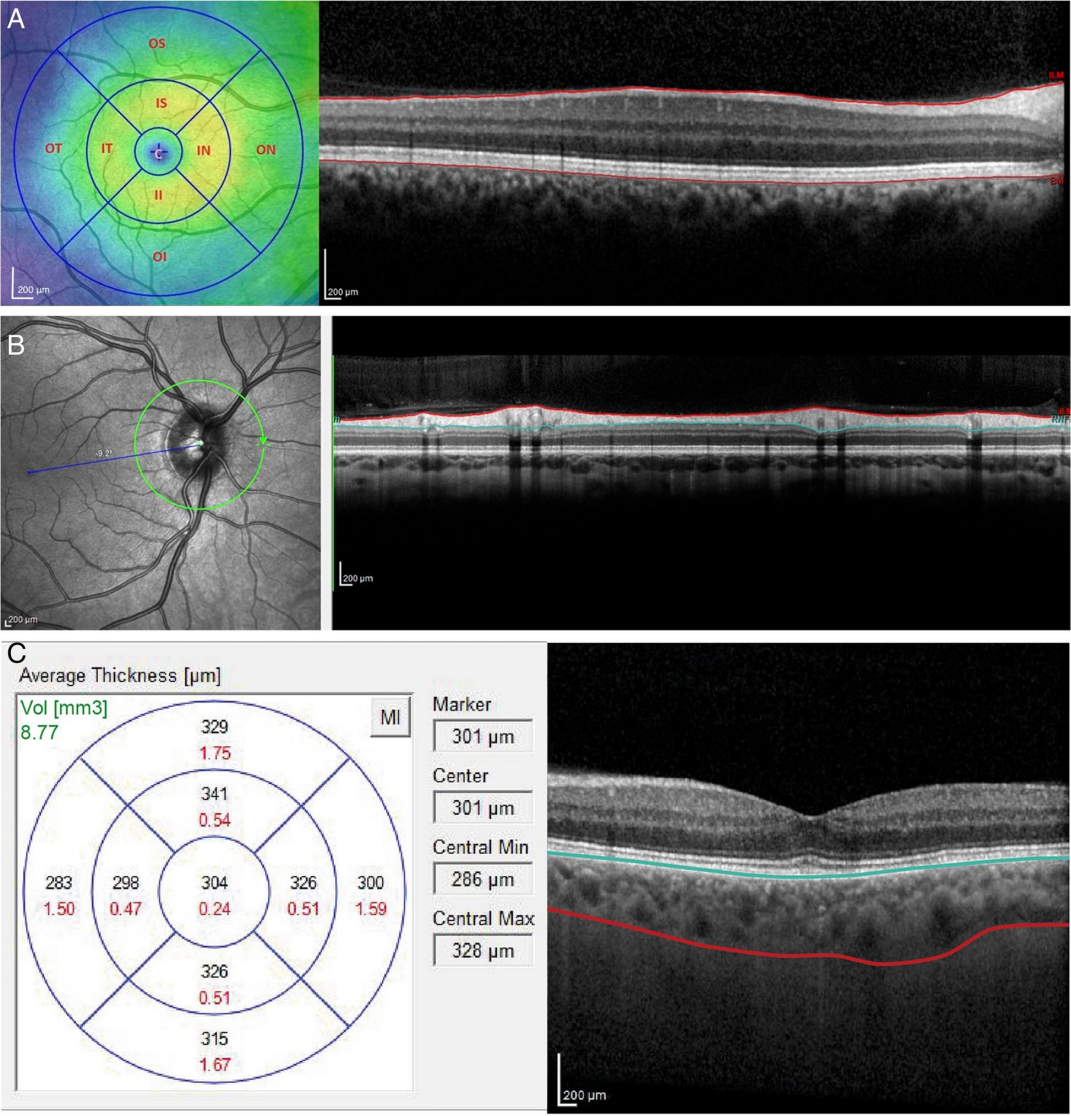
This figure shows the chorioretinal structures En face (left images) and as a cross section (right images). The Early Treatment Diabetic Retinopathy Study map divides the macula into 9 subfields. A circular grid is centered over the fovea and consists of 3 concentric rings of diameters 1, 3, and 6 mm, respectively. **A** The inner and outer rings are further divided into quadrants: temporal, nasal, superior, and inferior (left). Retinal thickness was defined as the area between the internal limiting membrane (ILM) and the inner boundary of retinal pigment epithelium (RPE) (right). **B** Retinal nerve fiber layer thickness was defined as the area bordered in red. **C** Choroidal thickness was measured as the perpendicular distance between hyperreflective outer border of the retinal pigment epithelium bruch’s membrane layer and the sclera-choroidal interface

Each examination comprised three scan protocols for each eye:

1. *A macular cube scan* was performed which consisted of 61 horizontal B scans with a 120 μm separation covering the entire macular area. Retinal thickness measurements were conducted using the protocol from the Early Treatment Diabetic Retinopathy study (ETDRS). The ETDRS map divides the macula into 9 zones, with a circular grid centered on the fovea and consisting of 3 concentric rings of diameters 1, 3 and 6 mm respectively. These rings are further divided into quadrants: temporal, nasal, superior and inferior. The retinal thickness was determined as the area between the internal limiting membrane (ILM) and the inner boundary of the retinal pigment epithelium. (RPE) (Fig.1a).
2. *A peripapillary circular line scan* centered over the optic disc. The 3.4 mm peripapillary scan mode was used to measure peripapillary RNFL thickness and to display the (temporal, superior, nasal, inferior and temporal, TSNIT) curve of RNFL thickness (Fig.1b).
3. *Choroidal thickness:* EDI mode was activated for all patients followed by manual segmentation of the choroid. Choroidal thickness was measured in all sectors of ETDRS grid as the perpendicular distance between hyperreflective outer border of the RPE-Bruch’s membrane layer and the choroido-scleral interface (Fig.1c).

Data were analyzed using IBM SPSS software package version 20.0. (Armonk, NY: IBM Corp). Qualitative data were described using number and percent. The Kolmogorov-Smirnov test was used to verify the normality of distribution. Quantitative data were described using range (minimum and maximum), mean, standard deviation (SD), median, and interquartile range (IQR). Student’s t-test and Analysis of Variance (ANOVA) were used to compare the mean values of the study groups. Correlations between different parameters were assessed using Pearson’s correlation. *P* values less than 0.05 was considered statistically significant.

## Results

This study included 144 eyes from 72 patients, with a gender breakdown of 53 male and 19 female participants, and an age range of 18 to 68 years old. The diagnosis of the cause of CKD was identified based on a combination of medical history, clinical examination, and various investigations including laboratory tests, renal ultrasound, and renal biopsy. The study cohort was made up of 28 patients with hypertensive nephrosclerosis, 19 patients with chronic glomerulonephritis, 9 patients with obstructive uropathy, seven patients with lupus nephritis, three patients with adult polycystic kidney disease, two patients with myeloma kidney, two patients with immunoglobulin-A (Ig-A) nephropathy, and two patients with an unknown etiology of CKD.

The mean IOP in the study participants was 16.7 mmHg, while the mean LogMAR value of BCVA was 0.13 ± 0.13. Approximately 38.9% of the participants were taking antihypertensive medication. A positive CRP was found in 33.3% of the participants, with a mean value of 35.75 ± 28.77 and a mean ESR level of 59.69 ± 34.72. The mean serum creatinine level was 3.86 ± 2.17 and the mean serum albumin level was 2.99 ± 0.85. The urine analysis showed albuminuria in 62.5% of the patients, with + 1 albuminuria in 15.3%, + 2 in 20.8%, and + 3 in 26.4%. The mean eGFR was 31.04 ± 24.83, and the patients were divided into three groups based on the stage of CKD: group 1 (CKD stage 1–2) included 11 patients, group 2 (CKD stage 3) included 19 patients, and group 3 (CKD stage 4–5) included 42 patients as shown in Table 1.

**Table 1:** Participant characteristics

Studied variables	Value
Age, years, mean (SD)	45.58 (16.12)
Male, n (%)	53 (73.6)
IOP (mean)	16.7 mmHg
Hypertension, n (%)	28 (38.9)
MAP (mean ± SD)	90 ± 5.85
BMI (mean ± SD)	24.7 ± 3.96
eGFR, ml/min/1.73m^2^, mean (SD)	31.04 (24.83)
CKD stage, n (%)	
1	4 (5.6)
2	7 (9.7)
3	19 (26.4)
4	18 (25)
5	24 (33.3)
Creatinine, mg/dl, mean (SD)	3.86 (2.17)
Serum albumin, mg/dl, mean (SD)	2.99 (0.85)
Hemoglobin, g/dl, mean (SD)	10.23 (2.31)
CRP, mean (SD)	35.75 (28.77)
ESR, mean (SD)	59.69 (34.72)
Albuminuria, n (%)	
Nill	27 (37.5)
+ 1	11 (15.3)
+ 2	15 (20.8)
+ 3	19 (26.4)

*SD* Standard deviation, *eGFR* Estimated glomerular filtration rate, *CRP* C reactive protein, *ESR* Erythrocyte sedimentation rate, *IOP* Intraocular pressure

Regarding retinal thickness, compared to those with earlier stages of CKD (stages 1–2), individuals with advanced stages of CKD (stages 4–5) had significantly lower mean retinal thickness values the central/foveal segment (*p* = 0.015), inner temporal segment (*p* = 0.023), outer temporal segment (*p* = 0.046) and inner nasal segments (*p* < 0.001). No significant differences in retinal thickness were observed between CKD categories for the superior, inferior, and outer nasal segments. Additionally, individuals in earlier stage CKD (stage 3) also showed lower retinal thickness in the central/foveal segment (*p* = 0.022) and outer temporal segment (*p* = 0.027). Table 2 plots in detail the obtained thickness measurements.

**Table 2:** Relation between CKD stages and Retinal thickness (*n* = 144)

Macular thickness	CKD stages			F	***P***
	Stage 1–2 (***n*** = 22)	Stage 3 (***n*** = 38)	Stage 4–5 (***n*** = 84)		
**Central thickness**					
Min. – Max.	254.0–288.0	249.0–287.0	226.0–304.0	4.455^*^	0.013^*^
Mean ± SD.	273.82 ± 10.67	262.58 ± 10.97	263.29 ± 18.19		
Median (IQR)	277.50(266.0–282.0)	258.0(256.0–266.0)	264.0(250.0–277.5)		
**Sig. bet. Grps.**	p_1_ = 0.022^*^,p_2_ = 0.015^*^,p_3_ = 0.971				
**Inner Nasal**					
Min. – Max.	273.0–337.0	302.0–351.0	295.0–350.0	8.266^*^	< 0.001^*^
Mean ± SD.	306.73 ± 14.51	315.66 ± 12.80	318.65 ± 11.37		
Median (IQR)	307.0 (302.0–313.0)	311.5 (306.0–323.0)	318.0 (310.0–327.0)		
**Sig. bet. Grps.**	p_1_ = 0.020^*^,p_2_ < 0.001^*^,p_3_ = 0.426				
**Inner Superior**					
Min. – Max.	297.0–345.0	263.0–350.0	268.0–352.0	1.597	0.206
Mean ± SD.	312.36 ± 13.45	310.55 ± 18.11	316.07 ± 16.46		
Median (IQR)	310.5 (300.0–320.0)	309.0 (303.0–320.0)	317.0 (307.5–325.0)		
**Inner Temporal**					
Min. – Max.	245.0–332.0	253.0–347.0	241.0–335.0	3.879^*^	0.023^*^
Mean ± SD.	305.27 ± 19.96	311.26 ± 15.60	302.24 ± 19.15		
Median (IQR)	309.0 (301.0–318.0)	313.5 (303.5–320.5)	301.0 (297.0–314.0)		
**Sig. bet. Grps.**	p_1_ = 0.320,p_2_ = 0.790,p_3_ = 0.023^*^				
**Inner Inferior**					
Min. – Max.	299.0–395.0	290.0–356.0	274.0–358.0	0.713	0.492
Mean ± SD.	316.45 ± 19.99	312.32 ± 15.78	315.74 ± 14.96		
Median (IQR)	311.5 (305.0–315.0)	309.5 (301.0–318.0)	314.0 (307.0–324.0)		
**Outer Nasal**					
Min. – Max.	237.0–322.0	251.0–323.0	259.0–338.0	1.256	0.288
Mean ± SD.	286.55 ± 20.28	292.97 ± 15.67	292.29 ± 15.55		
Median (IQR)	288.5 (280.0–296.0)	293.0 (287.0–304.0)	292.0 (285.0–301.5)		
**Outer Superior**					
Min. – Max.	263.0–353.0	218.0–322.0	240.0–385.0	1.148	0.320
Mean ± SD.	288.73 ± 22.38	287.68 ± 21.27	293.86 ± 23.48		
Median (IQR)	280.0 (271.0–300.0)	291.0 (281.0–300.0)	297.5 (280.0–303.0)		
**Outer Temporal**					
Min. – Max.	256.0–312.0	233.0–297.0	235.0–302.0	3.497^*^	0.033^*^
Mean ± SD.	285.41 ± 16.23	274.45 ± 14.19	277.40 ± 16.12		
Median (IQR)	284.0 (273.0–301.0)	274.0 (269.0–281.0)	279.0 (264.5–292.0)		
**Sig. bet. Grps.**	p_1_ = 0.027^*^,p_2_ = 0.046^*^,p_3_ = 0.599				
**Outer inferior**					
Min. – Max.	265.0–320.0	265.0–315.0	248.0–322.0	0.683	0.507
Mean ± SD.	286.59 ± 17.36	283.13 ± 12.14	282.14 ± 16.95		
Median (IQR)	285.0 (270.0–300.0)	284.0 (275.0–291.0)	283.5 (267.0–297.0)		

p1: *p* value for comparing between Stage 1–2 and Stage 3. p2: *p* value for comparing between Stage 1–2 and Stage 4–5. p3: *p* value for comparing between Stage 3 and Stage 4–5

^*^significant *p* value

**Fig. 2:**
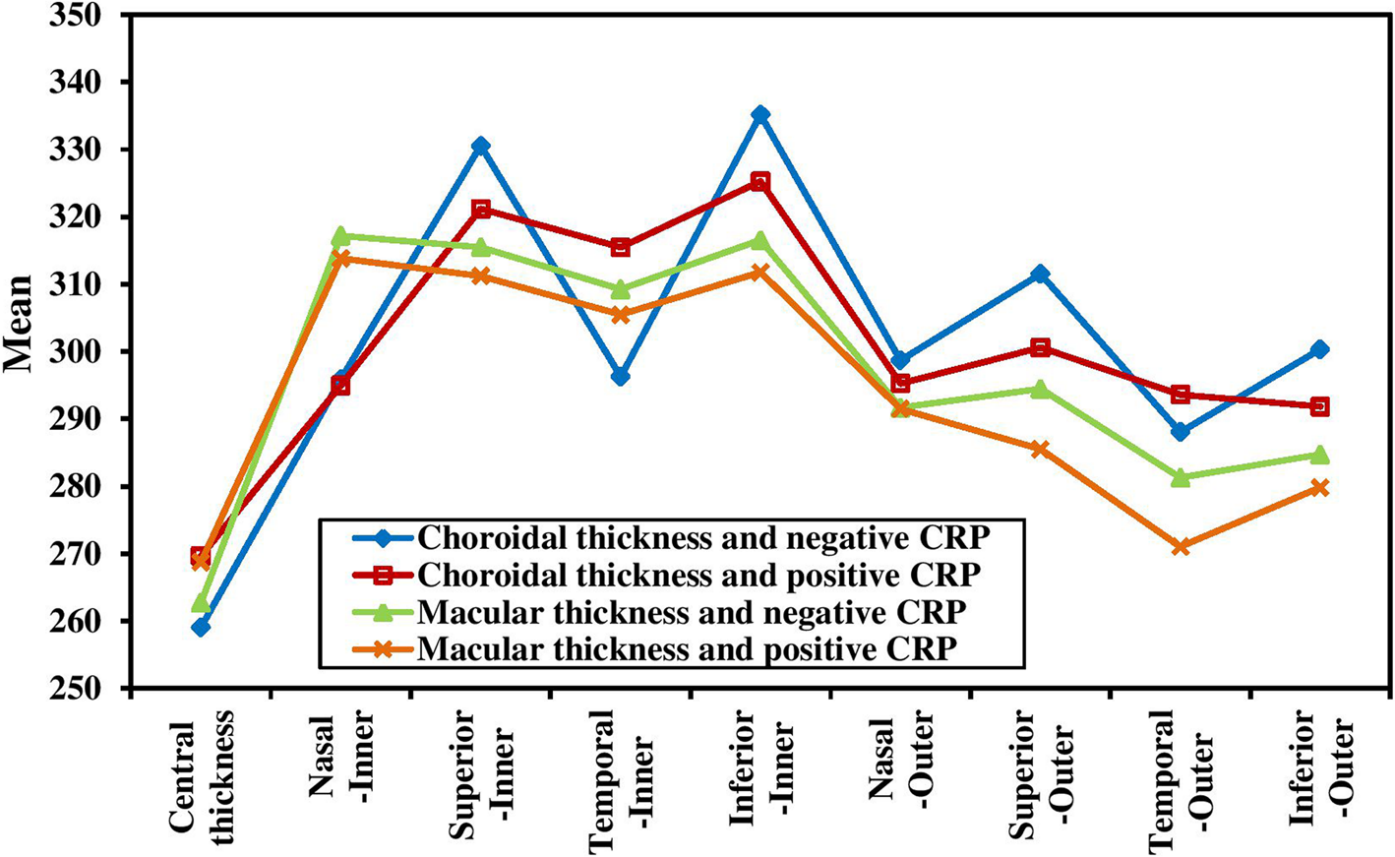
Relation between CRP and choroidal thickness and macular thickness (*n* = 144). CT = Choroidal thickness - MT = Macular thickness

**Fig. 3:**
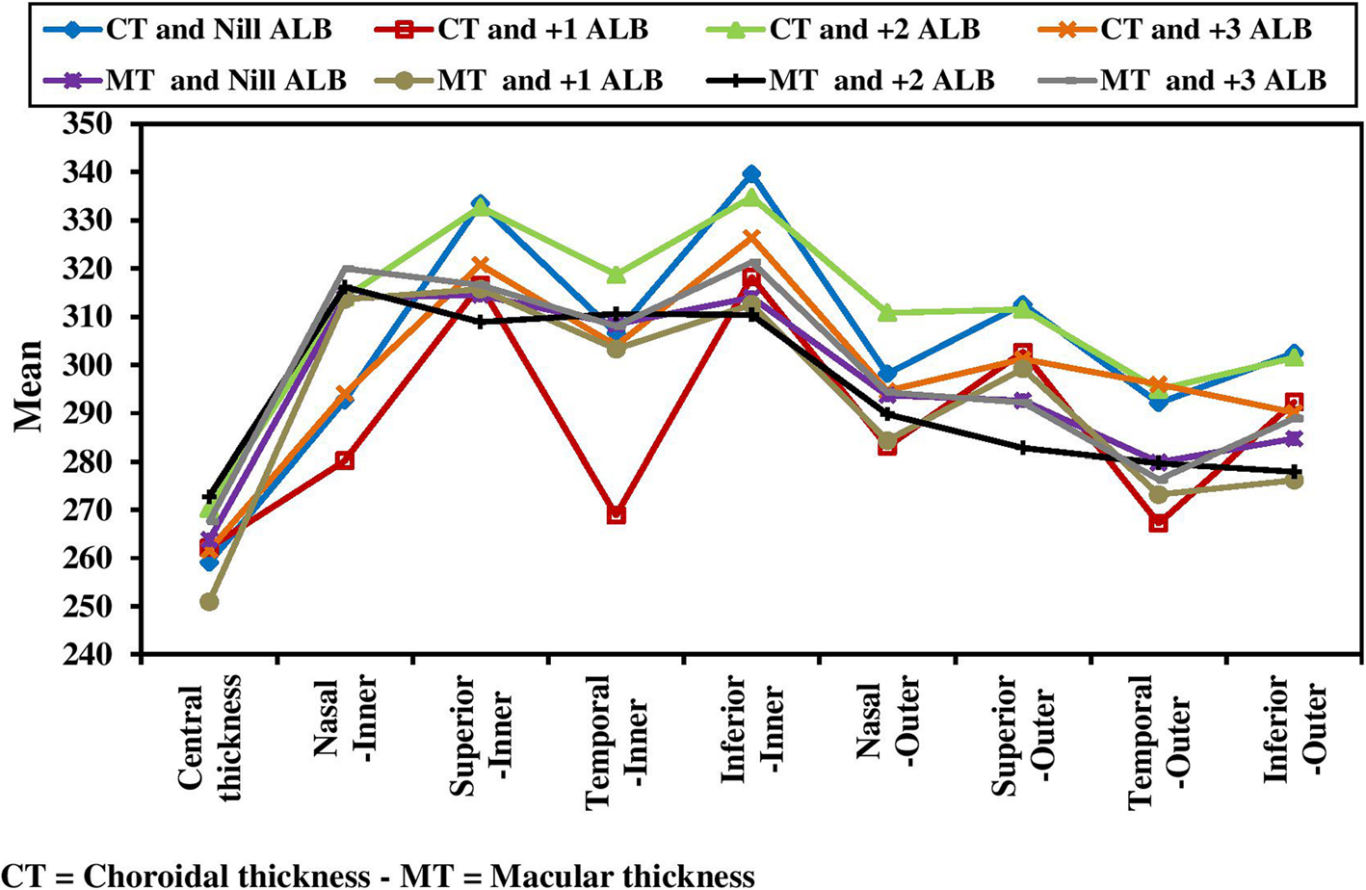
Relation between albuminuria and choroidal thickness and macular thickness (*n* = 144)

The study found that retinal thickness was independent from hypertension (HTN) status of the patients in all ETDRS regions. However, a negative correlation was observed between retinal thickness and CRP in certain areas of the eye, including the central/foveal segment (*P* = 0.032), outer superior segment (*P* = 0.005) and outer temporal segment (*P* < 0.001). This suggests that as CRP levels increase, retinal thickness in these areas decreases (Fig. 2). A negative correlation was also found between retinal thickness and albuminuria in the central/foveal segment (*P* < 0.001) and inner and outer inferior segments (*P* = 0.027 and 0.004 respectively) (Fig. 3). These findings suggest that the higher CRP and albuminuria levels, the more retinal thinning in certain areas.

For choroidal thickness, we found that advanced stages of CKD (stages 4–5) had significantly lower mean choroidal thickness in the central/foveal segment (*p* < 0.001), superior segment (*p* < 0.001), inferior segment (*p* < 0.001), inner nasal segment (*p* = 0.016) and inner temporal segment (*p* = 0.034) compared to earlier stages of CKD (stages 1–2). No significant differences in choroidal thickness were observed between CKD categories only for outer nasal and outer temporal segments. Additionally, individuals in earlier stage CKD (stage 3) also showed lower choroidal thickness in the central/foveal segment (*p* < 0.001), superior segment (*p* < 0.001), inferior segment (*p* < 0.001) and inner temporal segment (*p* = 0.045) as shown in Table 3.

**Table 3:** Relation between CKD stages and Choroidal thickness (*n* = 144)

Choroidal thickness	CKD stages			F	***P***
	Stage 1–2 (***n*** = 22)	Stage 3 (***n*** = 38)	Stage 4–5 (***n*** = 84)		
**Central thickness**					
Min. – Max.	250.0–346.0	164.0–298.0	202.0–393.0	31.079^*^	< 0.001^*^
Mean ± SD.	312.50 ± 20.47	238.26 ± 27.15	260.51 ± 41.08		
Median (IQR)	315.0 (300.0–324.0)	234.0 (229.0–249.0)	250.0 (232.0–283.0)		
**Sig. bet. Grps.**	p_1_ < 0.001^*^,p_2_ < 0.001^*^,p_3_ = 0.005^*^				
**Inner Nasal**					
Min. – Max.	285.0–346.0	220.0–359.0	191.0–382.0	4.189^*^	0.017^*^
Mean ± SD.	314.86 ± 20.53	299.68 ± 27.60	288.65 ± 46.47		
Median (IQR)	314.5 (296.0–340.0)	300.5 (291.0–316.0)	286.5 (267.5–314.5)		
**Sig. bet. Grps.**	p_1_ = 0.320,p_2_ = 0.016^*^,p_3_ = 0.323				
**Inner Superior**					
Min. – Max.	316.0–373.0	300.0–360.0	211.0–351.0	24.952^*^	< 0.001^*^
Mean ± SD.	358.55 ± 15.28	325.24 ± 18.55	320.21 ± 25.84		
Median (IQR)	364.0 (361.0–365.0)	322.0 (309.0–344.0)	321.0 (312.0–340.0)		
**Inner Temporal**					
Min. – Max.	300.0–344.0	221.0–329.0	177.0–433.0	3.573^*^	0.031^*^
Mean ± SD.	325.59 ± 12.07	297.24 ± 24.48	299.12 ± 54.43		
Median (IQR)	327.5 (317.0–334.0)	301.5 (286.0–313.0)	300.0 (266.0–324.0)		
**Sig. bet. Grps.**	p_1_ = 0.045^*^,p_2_ = 0.034^*^,p_3_ = 0.974				
**Inner Inferior**					
Min. – Max.	317.0–375.0	307.0–358.0	299.0–371.0	26.790^*^	< 0.001^*^
Mean ± SD.	356.36 ± 16.08	330.21 ± 16.85	326.15 ± 17.80		
Median (IQR)	360.0 (354.0–370.0)	326.0 (314.0–346.0)	322.0 (313.5–339.5)		
**Outer Nasal**					
Min. – Max.	256.0–344.0	247.0–318.0	230.0–469.0	2.728	0.069
Mean ± SD.	295.23 ± 29.14	286.87 ± 20.88	302.95 ± 41.73		
Median (IQR)	292.0 (268.0–326.0)	293.5 (269.0–305.0)	297.5 (273.0–317.0)		
**Outer Superior**					
Min. – Max.	301.0–347.0	281.0–323.0	244.0–357.0	30.885^*^	< 0.001^*^
Mean ± SD.	334.68 ± 14.99	303.53 ± 12.03	302.81 ± 19.81		
Median (IQR)	341.0 (339.0–342.0)	303.0 (294.0–317.0)	304.0 (296.0–306.0)		
**Outer Temporal**					
Min. – Max.	275.0–340.0	253.0–311.0	230.0–403.0	2.862	0.061
Mean ± SD.	301.86 ± 16.45	285.11 ± 15.33	288.90 ± 32.33		
Median (IQR)	300.5 (289.0–305.0)	287.5 (272.0–299.0)	286.5 (266.0–312.5)		
**Outer Inferior**					
Min. – Max.	299.0–331.0	266.0–339.0	251.0–358.0	20.590^*^	< 0.001^*^
Mean ± SD.	321.55 ± 10.42	293.63 ± 14.38	292.92 ± 22.39		
Median (IQR)	325.0 (324.0–327.0)	297.5 (284.0–300.0)	294.0 (273.5–306.5)		

^*^significant *p* value

Furthermore, choroidal thickness was negatively associated with hypertension (HTN) only in the outer inferior segment (*p* = 0.003). A negative correlation was also observed between choroidal thickness and CRP in the superior segment (*p* = 0.012 for inner superior segment and < 0.001 for outer superior segment), inferior segment (*p* < 0.001 for inner inferior segment and 0.025 for outer inferior segment) and inner temporal segment (*p* = 0.023) (Fig. 2). Similarly, choroidal thickness was negatively associated with albuminuria in all ETDRS segments except the central/foveal segment (Fig. 3).

In contrast to retinal and choroidal thicknesses, RNFL thickness was not significantly different among the three groups studied in any ETDRS segment as shown in Table 4.

**Table 4:** Relation between CKD stages and RNFL (*n* = 144)

RNFL	CKD stages			F	***P***
	Stage 1–2 (***n*** = 22)	Stage 3 (***n*** = 38)	Stage 4–5 (***n*** = 84)		
**Nasal**					
Min. – Max.	63.0–116.0	49.0–93.0	49.0–131.0	2.850	0.061
Mean ± SD.	81.64 ± 13.35	72.45 ± 10.04	78.10 ± 17.74		
Median (IQR)	80.50 (73.0–85.0)	73.0 (66.0–78.0)	70.0 (65.50–89.0)		
**Superior**					
Min. – Max.	110.0–168.0	110.0–159.0	102.0–180.0	0.155	0.857
Mean ± SD.	128.32 ± 14.62	127.58 ± 10.81	126.56 ± 15.98		
Median (IQR)	123.5 (117.0–135.0)	127.0 (118.0–138.0)	123.0 (116.0–133.5)		
**Temporal**					
Min. – Max.	63.0–100.0	50.0–104.0	49.0–176.0	1.610	0.204
Mean ± SD.	76.86 ± 11.54	76.0 ± 13.18	82.20 ± 23.11		
Median (IQR)	76.0 (66.0–84.0)	73.50 (67.0–85.0)	78.0 (66.0–91.0)		
**Inferior**					
Min. – Max.	112.0–148.0	95.0–141.0	64.0–168.0	2.445	0.090
Mean ± SD.	128.45 ± 9.80	123.61 ± 10.53	121.26 ± 15.71		
Median (IQR)	125.0 (122.0–137.0)	124.50 (118.0–132.0)	121.0 (112.5–128.0)		

## Discussion

In our study, we examined the relationship between thickness of the chorioretinal layers measured using SD-OCT and the stages CKD in 72 patients with 144 eyes. We found that thinner retina and choroid were associated with CKD stage 3 and even more thinning in CKD stage 4–5. There was no association found between retinal and choroidal thickness and hypertension. However, we did observe associations between CRP and albuminuria, which were inversely correlated with choroidal thickness in CKD and were independent predictors of thickness. These findings may be explained by the role of inflammation in the development of both vascular and renal disease.

In this study, it was found that the choroidal thinning is more pronounced in advanced CKD. This thinning may be a reflection of a direct microvascular insult. As the vessels of the choroid supply the outer retinal layers and the RPE, it is possible that retinal abnormalities may occur as a result of choroidal pathology. The choroid plays a vital role in retinal function, as it nourishes the outer half of the retina. Abnormal choroidal circulation can lead to retinal photoreceptor dysfunction and death. Many diseases such as central serous retinopathy (CSR), age-related macular degeneration (AMD), pathological myopia and Vogt-Koyanagi-Harada (VKH) disease are related to choroidal changes. Identifying choroidal changes accurately will enable proper assessment of many posterior segment diseases. However, unlike the retina, choroidal structures do not have distinct, ordered layers and lack contrasting reflective properties, making it difficult to examine the choroid in as much detail as the retina [6]. Additionally, it is suggested that the disturbance of the autonomic nervous system may contribute to the changes observed. CKD is associated with increased sympathetic activity, which may play a role in the progression of the disease. While the choroidal circulation has autonomic innervation, the retinal circulation does not, thus the thinning of the outer retina and choroid might be related to increased sympathetic tone affecting the choroidal vasculature [7]. However, the study did not investigate the measurement of sympathetic activity.

Maria Vadalà and colleagues [7] conducted a study using swept source optical coherence tomography (SS-OCT) and optical coherence tomography angiography (OCTA) scans of the macular region to examine the association between retinochoroidal parameters and renal impairment in hypertensive, non-diabetic patients. They found that CKD is associated with thinning of both the retina and choroid, decrease in vascular density in both the superficial and deep perifoveal network, an inverse relationship between albuminuria and thickness of both the choroid and retina, and no correlation between blood pressure measurements and chorioretinal thicknesses and vascular density assessed by OCTA. Similarly, Ling Yeung and colleagues [8] assessed early retinal microvascular changes in patients with CKD via OCTA and reported significant retinal microvascular abnormalities such as blunt ended vessels, increased vascular tortuosity, and localized non-perfusion areas in patients with CKD.

In end stage renal disease, Ali Kal and colleagues [9] found that hemodialysis leads to a significant decrease in choroidal thickness, while there is no change in retinal thickness. This might be due to the extensive fluid absorption, which could affect ocular blood flow. A cross-sectional study conducted by Boem Changand et al. [10] also found a significant reduction in choroidal thickness at the macula and outside the macula, and this decrease in choroidal thickness was correlated with body weight loss, similar to the findings of Yang et al. [11]. The goal of hemodialysis is to maintain the kidneys’ excretory functions in ESRD patients, but during a hemodialysis session, ultrafiltration removes excess fluid from plasma, which leads to depletion of blood volume and an increase in the plasma protein concentration (rise of plasma colloid osmotic pressure), and a decrease in serum osmolality. This volume depletion is compensated by vascular refilling from the interstitial and intracellular space. As the choroid has a rich vascular network and the highest blood supply in the eye, these changes might affect its architecture [12]. In contrary to our study, Paterson and colleagues [1] reported that no significant associations were detected between choroidal volume or choroidal vascular index (CVI) and CKD, while the decrease in retinal thickness, particularly a thinner inner retinal layer, was found to be associated with CKD stage 4–5. Wu and colleagues [13] investigated retinal, neural and microvascular changes in different stages of CKD (stage 3–5) by SD-OCT and OCTA, and reported that retinal neural impairment was associated with decreased eGFR and more advanced CKD stage.

In our study, we found that there was no difference in Retinal nerve fiber layer (RNFL) thickness between the groups studied. This is in agreement with the findings of Craig Balmforth and colleagues [4], who conducted a study involving 150 subjects, including 50 patients with hypertension, 50 with CKD, and 50 matched healthy controls. They reported that retinal thickness, macular volume, and choroidal thickness were all reduced in CKD compared with hypertensive and healthy subjects. They also found that thinner choroid was associated with a lower estimated glomerular filtration rate (eGFR) and, in CKD, with proteinuria as well as increased circulating C-reactive protein, interleukin 6 (IL-6), asymmetric dimethylarginine (ADMA), and endothelin-1 (ET-1). However, Demir and colleagues [14] documented significant RNFL thinning in CKD without DM. Jung and colleagues [15] also reported reduction of thickness in the temporal and superior sectors of RNFL scan in patients with ESRD.

There were several limitations in this study. First, the medications taken by our patients, such as angiotensin-converting enzyme inhibitors, β-blockers, and statins may have had effects on the OCT parameters studied. However, all patients were stabilized on their therapies and this was an unavoidable limitation of such studies. Secondly, the lack of any angiographic examination which can study in deep the retinal and choroidal vascular structure like indocyanine green (ICG), fluorescein fundus angiography (FFA) and optical coherence tomography angiography (OCTA) which can study in deep the retinal and choroidal vascular structure. Also, the cross-sectional nature of this study does not allow for the determination of causality of association. Saying that, the clinical relevance of these findings requires further consideration through longitudinal evaluation of changes in retinal thickness with declining kidney function with age. Lastly, the specificity of the observed associations with the underlying cause of CKD was not examined. Longitudinal studies are needed to establish the chronological correlation with CKD severity. As well, focused studies on CKD subgroups based on the underlying pathology.

## Conclusion

This study revealed that chorioretinal thinning in CKD is associated with a lower eGFR, a higher serum CRP, and greater proteinuria. These findings support the potential use of chorioretinal thickness profile as a biomarker for CKD progression.

## Data Availability

The data sets used and/or analyzed during the current study are available from the corresponding author on reasonable request.
